# Junior Students and Recruiting

**Published:** 1915-11-13

**Authors:** 


### The Hospital

The Workers' Newspaper of Administrative Medicine and Institutional Life, Administration, National Insurance and Health.

No. 1535, Vol. LIX. SATURDAY, NOVEMBER 13, 1915. PRICE ONE PENNY.

### JUNIOR STUDENTS AND RECRUITING.

The junior student of medicine has during recent months been in a position to realise that in the multitude of counsellors there is apt to be confusion rather than safety; and even yet the stream of amateur advice has not exhausted itself. Fortunately, those who have the right to speak with authority and who are in a position to review the position from all possible points of view have now given a clear utterance, and the student will be well advised to listen to this rather than to suggestions which own a mere individual value. It is quite true that the War Office has changed its opinion, and that in some quarters this is urged as a reproach against the authorities. But it may be mildly remarked that the circumstances have changed, and that the needs of the nation and the duty of the individual citizen are not exactly the same as they were deemed to be some six to twelve months ago. What is essential at the moment is a steady supply of young men to maintain and reinforce the armies in the field, and to this other claims must yield, even though in themselves they are important and beyond question. The position is one in which the "perspective" recently recommended to the nation must be cultivated and the urgent must be preferred to the important. No doubt it is an unpleasing prospect to realise that some years hence there is likely to be a short supply of doctors for civil practice. Equally it is unpleasing to realise that we are engaged in a fight for our national existence. The fact, however, remains, and it is a fact which governs all our immediate arrangements. It will undoubtedly be an unfortunate thing to find that during the next few years our people and the public health services may be prejudiced for lack of a full measure of medical knowledge and skill. But the position will be much more unfortunate should the Germans win the war and impose their will upon doctors and laymen alike. To prevent such a result is the first demand, and when this is accomplished the difficulties of the future, however inconvenient they may be, must be met by the resources of the future.

There is no longer any doubt of the opinion of the authorities, and, indeed, it is difficult to understand how any doubt could exist since the statement made some three months ago that the War Office was "unwilling to suggest that junior students should be discouraged from taking combatant commissions." This, as The Hospital interpreted it in its issue of September 11, could only mean that in a considered decision "the immediate necessities of the Army must take precedence of the more remote claims of the civil population." The conclusion was challenged in certain quarters, but the event has proved that our reading was justified. Now we have both from the War Office and from the Director-General of Recruiting a quite unambiguous definition that junior medical students of military age occupy relative to the Army exactly the same position as that of other citizens eligible for military service. Each one of these, therefore, has to face the question whether he ought or ought not to answer to the country's call; and to his own conscience he must stand or fall. All that it is necessary to add is that while for some there may be, as in other classes, physical or other disqualifications which render an affirmative answer impossible, for none is there any longer any competition in the suggestion that his duty is to complete his medical studies and obtain a diploma entitling him to practise. On this point, not quite excusably as we think, there has been some doubt and uncertainty. Now all this is ended, and it may be hoped that medical teachers and others who may doubt the wisdom of the decision will recognise that it is their duty to conceal their private judgments and to allow the judgment of the authorities to have a full opportunity. Of the personal inclination of the students themselves, at least in the great majority of cases, there can be no doubt, and many will heartily rejoice at the removal of a hindrance which they felt to be a substantial one. They are now to have a freedom which is denied to their seniors, for while the claim on the juniors is for active service, not less distinct is the demand that students who are within two years of qualification shall remain at home and complete the curriculum. That some of the seniors are restive under this demand is easily understood, but the time is one for obedience. Let the young men remember the veteran Field-Marshal who has expressed his readiness to sweep the crossing as soon as the Government gives him the order to take up that employment.

The need for presenting the actual position of the medical student in relation to the recruiting effort now being conducted under the direction of Lord Derby arises, in the first place, from the uncertainties which have attended the question, and to which allusion has already been made. It arises, in the second place, from the fact that even at the present moment there are those who propose openly to question the wisdom of the course now recommended. The alternative proposal advanced is that the medical curriculum should be made to include as one of its compulsory features membership of an Officers' Training Corps, and that the student presumably should both continue his studies and pursue his military training. Such an arrangement may, of course, be quite possible in time of peace when the necessary drill and discipline can be pursued in an interrupted and leisurely fashion. But in existing circumstances the combination is for all practical purposes useless. If the student is to be fitted for the position of an officer in the course of a few months, he will certainly have no time for the medical curriculum, and an attempt to combine the two must inevitably mean that while the officer is late in appearing the doctor is not substantially advanced. But what the situation requires is a prompt supply of combatants, and these can only be secured by undivided devotion to military training. The effort to sit on two stools at one and the same time is proverbially disastrous, and there is no reason to expect success in the present instance. The position is made perfectly clear by the official announcement, and there it seems necessary to leave it.

